# People Are Taller in Countries With Better Environmental Conditions

**DOI:** 10.3389/fendo.2020.00106

**Published:** 2020-03-11

**Authors:** Alina German, Gustavo Mesch, Ze'ev Hochberg

**Affiliations:** ^1^Pediatric Department, Bnei-Zion Medical Center, Haifa, Israel; ^2^Rappaport Family Faculty of Medicine, Technion—Israel Institute of Technology, Haifa, Israel; ^3^Department of Sociology, University of Haifa, Haifa, Israel

**Keywords:** environment, growth, height, stress, inequality, social

## Abstract

**Background:** Height is considered an indicator of health and well-being of an individual and population. Height variation results from a complex interaction of genetic, environmental, socioeconomic, and cultural influences. In order to understand the contribution of environmental stress associated with the child's growth, we correlated indicators of a stressful environment with adult height.

**Methods:** We utilized seven equally weighted indicators of a stressful environment: homicide rates, GDP per capita, income inequality (GINI index), corruption perception index (CPI), unemployment rate, urban air pollution, and life expectancy (LE). Data on male and female height by country from 1992 to 1996 were obtained from the NCD Risk Factor Collaboration dataset. We assessed separately data from the 31 member countries of the Organization for Economic Co-operation and Development (OECD). In order to establish whether the indicators reflected a single conceptual dimension, we conducted an exploratory analysis and principal component analysis (PCA) with orthogonal transformation of the original variables. The relationships between male and female heights and the z-transformed principal components: Quality of life (QoL) and the Social factor (SF) that were derived after the PCA was assessed.

**Results:** Male and female heights strongly correlated (*p* < 0.0001) with each of the seven indicators. In the PCA, the indicators clustered into “Quality of Life” factors (QoL), which comprised the CPI, GDP, air pollution, LE, and “Social factors” (SF), which comprised homicide rate and GINI index. For males and females, the average height by country strongly correlated with QoL (*p* < 0.0001) and SF (*p* < 0.0001). Within OECD countries, male and female height strongly and negatively correlated with the SF, but not with QoL.

**Conclusion:** Growth attenuation is a tradeoff adaptive response: a calorie used for growth cannot be used for fighting stress. Here we show that: (1) Adult height, when used as a measure of child's growth, is an indicator of a stressful environment in context with the genetic background and spatial factors; (2) Stressful QoL factors and the SF exert a greater effect on men's height than women's height; and (3) The ranking of the indicators of short stature are income inequality > air pollution > GDP > CPI > homicide rate > LE > unemployment.

Height variation in the population of different countries and regions and within a specific population is the result of a complex interaction of genetic (1), environmental, socio-economic, and cultural factors (2), including parental education health and literacy (3). Importantly, adults stature is the result of longitudinal growth during childhood. Since height is considered to be an indicator of the health and well-being of an individual and a population, the variation in adult height can be used as an accurate marker of inequalities in human environments.

Millions of children worldwide not only fail to achieve their linear growth potential because of suboptimal health conditions and inadequate nutrition and care; they can also suffer severe irreversible physical and cognitive damage that accompanies stunted growth (4).

Much attention has been devoted to the contribution of nutrition in height and stunting (4). The long-term outcomes of stunting include a pure stature effect, and the effect of lost growth potential encompassing the cognitive impacts of undernutrition [A review of the evidence linking child stunting to economic outcomes (5)].

Stress is defined as a “state in which homeostasis is threatened or perceived to be so (6) and the spectrum of human stressors range from the daily hassles to starvation and bereavement (7).” Before modern times, the greatest stresses were infection, starvation, and malnutrition. These stressors of our predecessors have been replaced by pollution, poverty, gun violence, financial pressure, social and racial discrimination, and economic inequality. These stressors affect entire families, parents and children alike, and their effects depend on the type, intensity, timing, and duration of the exposure to stress.

Macroeconomic indicators of environmental stress include gross domestic product (GDP), income inequality, and unemployment. This economic and social context generates social inequality in access to health resources, social support, and healthy lifestyles. Differences in health outcomes, particularly in children, are the result of social inequality. Income inequality is often associated with increased homicide rates (8, 9). These indicators are social stressors that reduce interpersonal trust and can be associated with variation in levels of health, which includes post-traumatic stress disorder (10).

Another important dimension is the quality of governance effectiveness, and a state's ability to enforce its rules. There is empirical evidence showing that poor governance has a wide range of effects which include difficulties to provide food security and appropriate health services access to the population (11). Countries which score well on the quality of governance also tend to score better than other countries on poverty reduction, health care provision, and subjective well-being (12). A recent study has shown that low government efficiency and corruption have an impact on various indicators of child deprivation which include lack of safe drinking water, malnutrition, lack of access to health care, and lack of access to information (12). The article concluded that low-quality governance explains much of the cross-country variation in child deprivation.

The social stressful conditions, when they are experienced in early life, can profoundly influence child development, growth, and maturation and have long-term consequences on developing biological systems and long-term health (13–15). It was previously reported that children's growth can serve as an indicator of the extent to which social inequality exists in a population, as well as temporal changes in the economic condition of society as a whole and its specific subgroups (16).

There is an emerging notion that a stressful environment changes a child's gene expression and hormonal activity, and contributes to biological changes that may lead to mental and physical disorders (17).

In 2015, Bloomberg ranked the most stressed countries according to their living environments (http://www.bloomberg.com/visual-data/best-and-worst//most-stressed-out-countries). This ranking was based on seven indices, namely homicide rates, GDP per capita, income inequality, corruption perception index (CPI), unemployment rate, urban air pollution, and life expectancy.

Comparing vulnerability to environmental stress across countries can identify those leverage points where vulnerability and, by inference, social and emotional stress can be reduced at least in the short to medium term. Identification of particularly vulnerable nations or regions can act as an entry point for both understanding and addressing the processes that cause and exacerbate vulnerability. Since the impact of the economic and social indicators of stress on child growth and maturation have not been extensively studied, we aimed in this study to understand the impact of these indicators on average male and female height, as a measure of child growth in 71 countries.

## Methods

### Data Sources

We used GDP per capita, income inequality, life expectancy, CPI, unemployment rate, homicide rates, and urban air pollution as the indicators of a stressful environment.

For each indicator, we combined the average mean value for women and men by country for the period, 1990–2000.

Data on GDP per capita ($US) were extracted from the national accounts data of the World Bank and the national accounts data files of the Organisation for Economic Co-operation and Development (OECD) and entered into the database.

Income inequality or the GINI index measures the extent to which income distribution (among individuals or households within an economy or, in some cases, consumption expenditure) deviates from a perfectly equal distribution. Thus, a GINI index of 0 represents complete equality, while an index of 100 represents complete inequality. These data were collected from the World Bank dataset and the actual scores were entered into the database.

Life expectancy at birth indicates the number of years a newborn infant would live if prevailing patterns of mortality at the time of birth were to stay the same throughout life. These data were collected from the World Bank's data bank, whose sources are (a) the UN Population Division, World Population Prospects, (b) the United Nations Statistical Division, Population and Vital Statistics Report (various years), (c) census reports, and other statistical publications from national statistical offices, (d) Eurostat: Demographic Statistics, (e) Secretariat of the Pacific Community: Statistics and Demography Program, and (f) the US Census Bureau: International Database (http://data.worldbank.org/indicator/SP.DYN.LE00.IN) and entered into the database.

The CPI grades countries on the perceived level of public sector corruption by expert assessments and opinion surveys. The scale ranges from 0 to 100, where 0 means that a country is perceived as highly corrupt and 100 means it is perceived as very honest. The actual score was extracted from the website of the international non-governmental organization Transparency International (http://www.transparency.org/research/cpi/overview) and entered into the dataset.

Data on unemployment rate are given as percent of total labor force. It refers to the share of the labor force that is without work and seeking employment. These data were extracted from the World Bank's data bank whose sources were the International Labor Organization's database http://data.worldbank.org/indicator/SL.UEM.TOTL.ZS and entered into the database.

The average data by country from 1990 to 2000 on homicide rates in percent were extracted from the Global Study on Homicide, which was published by the UN Office on Drugs and Crime and based on the intentional homicide count and rate per 100,000 habitants according to country/territory (https://www.unodc.org/documents/congress/backgroundinformation/Crime_Statistics/Global_Study_on_Homicide_2011.pdf) and entered into the database.

Urban air pollution is expressed as the particulate matter concentration micrograms per cubic meter. Fine suspended particles whose diameters are <10 microns (PM10) are capable of penetrating deep into the respiratory tract, where they cause significant health damage. These data were extracted from the database of the Global Model of Ambient Particulates (GMAPS) (18) and entered into the database. Estimates represent the average annual exposure level of an average urban resident to outdoor particulate matter in residential areas of cities with more than 100,000 habitants.

Data on average male and female height by country from 1992 to 1996 were obtained with permission from the NCD Risk Factor Collaboration (NCD-RiSC) dataset (http://www.ncdrisc.org/d-height.html) and entered into the database. This dataset includes sources that were representative of a national, subnational, or community population and had measured height. Self-reported height and data sources on population subgroups whose anthropometric status may differ systematically from that of the general population were not included in the study (19).

We also compared the data on average male and female height as a function of stressful environment indicators from developed and developing countries and data from the 31 member countries of the OECD with those from non-OECD countries. South Korea, Switzerland, New Zealand, and Luxembourg at least one of the indicators of the stressful environment was missing and data from these countries was not included in the analysis.

### Statistical Methods

In the analysis, we used information on the average male and female height and the seven indicators of a stressful environment: GDP per capita, income inequality, life expectancy at birth, CPI, unemployment rate, homicide rates and urban air pollution from 71 countries.

All statistical analyses were done using software statistical package (IBM SPSS Statistics 20.0) and statistical significance was set as 5%. The strength of the linear relationship between the average male and female height per country and each indicator of a stressful environment was determined by calculating Pearson's correlation coefficient.

Since the bivariate correlations of the indicators of a stressful environment had a high value, we did an explorative principal component analysis (PCA) in to identify the extent that the indicators of a stressful environment reflect conceptually meaningful separate constructs.

A correlation matrix of orthogonal-transformed data was generated using a PCA (IBM SPSS Statistics 20.0). The procedure uses orthogonal transformation to convert our set of variables into a set of values of linearly uncorrelated variables.

We calculated the Pearson's correlation coefficient between our dependent variable (male and female heights for the71 countries) and the independent scales (QoL and SF). The bi-variate correlations were calculated separately for non-OECD and OECD countries using Spearman's correlation coefficient.

## Results

When we calculated the bivariate correlations of the individual variables, we found that male and female height was positively correlated with the CPI, life expectancy at birth, and GDP per capita and negatively correlated with homicide rates, income inequality, unemployment rate, and urban air pollution. Men and women were taller in those countries with a low CPI, a high life expectancy at birth, and a high GDP per capita than men and women in those countries with a high CPI, a low life expectancy at birth, and a low GDP per capita (Table 1).

**Table 1:** The Pearson's correlation coefficients between the seven indicators of environmental stress and male and female heights.

	**GDP per capita**	**Life expectancy**	**Urban air pollution**	**Unemployment rate**	**CPI**	**Income inequality**	**Homicide rate**
Male height	0.60^**^	0.62^**^	−0.51^**^	−0.30^*^	0.66^**^	−0.52^**^	−0.29^*^
Female height	0.52^**^	0.54^**^	−0.51^**^	−0.32^*^	0.61^**^	−0.52^**^	−0.26^*^

*GDP, gross domestic product; CPI, corruption perception index*.

* *p = 0.01*,

** *p < 0.001*.

**Figure 1:**
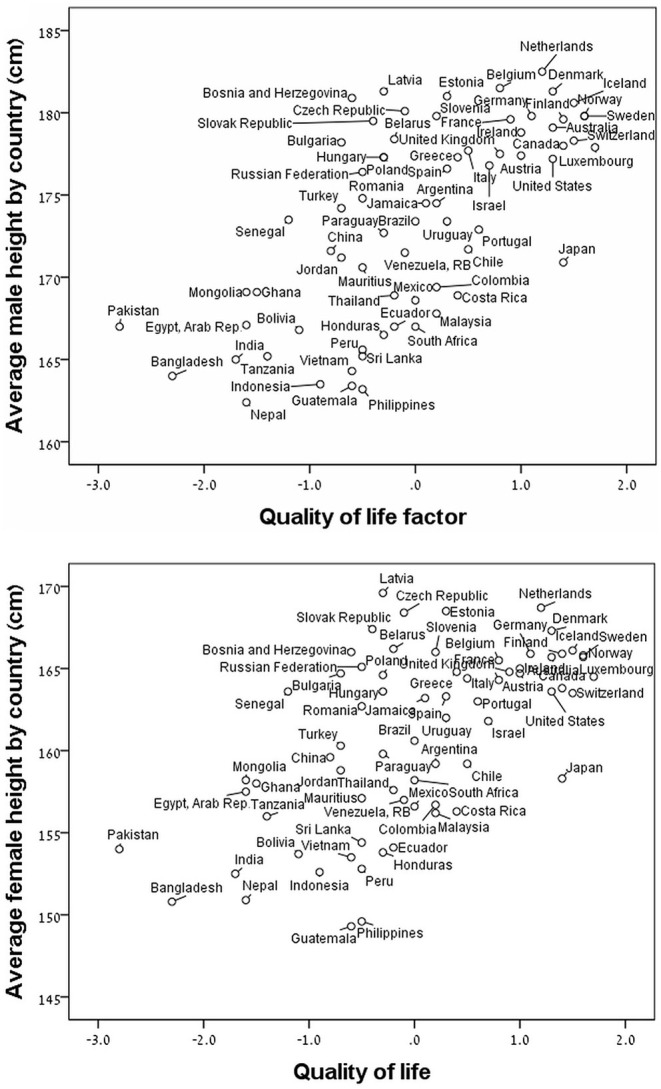
Average height by country as a function of the quality of life factor (Z-score) for males (upper panel; *r* = −0.65, *p* < 0.0001) and for females (lower panel; *r* = −0.60, *p* < 0.001).

**Figure 2:**
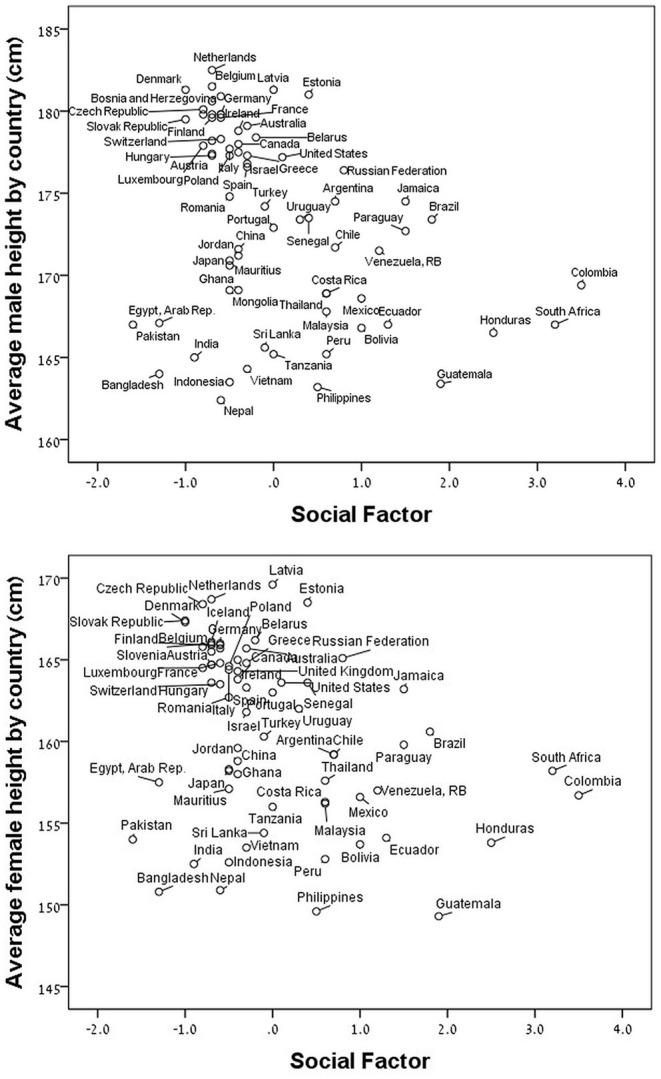
Average height by country as a function of the social factor (Z-score) for males (upper panel: *r* = −0.35, *p* < 0.0001) and for females (lower panel; *r* = −0.32 *p* < 0.005).

The results of the PCA indicated that the individual variables represent two different components according to the Eigenvalue and accounted for 77.6% of the total variance (Table 2). The first component, which we called “Quality of Life” (QoL), comprises the CPI, GDP per capita, urban air pollution, and life expectancy at birth, and accounts for 54.1% of the variance. The second component, which we called “Social Factor” reflects social problems conducive to social stress and comprises the homicide rate and the income inequality, and accounts for 23.6% of the variance. The unemployment rate did not fit into any of the other extracted factors because is not an independent dimension. Since the unemployment rate could be included in the QoL and social factor components it was not included in the analysis in order to reduce the risk of multicollinearity with the extracted independent factors. For males and females, the average height by country strongly and positively correlated with QoL (*p* < 0.0001) (Figure 1) and strongly and negatively correlated with SF (*p* < 0.0001) (Figure 2).

**Table 2:** Clustering of the stress determinants by principal component analysis.

**Stress indicators**	**QoL factor**	**SF factor**
CPI	0.903	
GDP per capita	0.895	
Life expectancy	0.818	
Urban air pollution	0.618	
Homicide rate		0.709
Income Inequality		0.696

*CPI, corruption perception index; GDP, gross domestic product*.

For the 40 non-OECD countries, we found that the average male and female heights by country were strongly and positively correlated with the QoL component and strongly and negatively correlated with the Social Factor component (Table 3). For the 31 member countries of the OECD, we found that the male and female heights were strongly and negatively correlated with the Social Factor component only (Table 3).

**Table 3:** Pearson's correlation coefficients of male and female height as a function of Quality of Life (QoL) and the social factors for the 31 member countries of the Organization for Economic Co-operation and Development (OECD) and the 40 non-OECD countries.

**Correlated variables**	**OECD countries *N* = 31**	**Non-OECD countries *N* = 40**
Male Height vs. Quality of Life	0.24	0.65^**^
Female Height vs. Quality of Life	0.05	0.60^**^
Male Height vs. Social Factor	−0.50^**^	−0.35^**^
Female Height vs. Social Factor	−0.48^**^	−0.32^**^

** *p < 0.001*.

We found that between-countries variations are similar to those within countries: for men, the between-countries average height ranges from 158 to 183 cm (Indonesia and Netherlands, respectively), with a standard deviation (SD) of 5.9 cm, as compared to 164–190 cm within the USA (3–97th percentile, SD = 6.5 cm) (20). For women, the between countries average height ranges from 147 to 170 cm (Indonesia and Netherlands, respectively, SD 5.7 cm), as compared to 151–175 cm within the USA (SD = 6.0 cm).

## Discussion

This report informs on the results of an investigation on the influence of the environment on a child's growth using average adult male and female heights in 71 countries as the ultimate measure of a child's growth. We found that men and women were taller in those countries with a low CPI, a high life expectancy at birth, and a high GDP per capita than men and women in those countries with a high CPI, a low life expectancy at birth, and a low GDP per capita. Obviously, there is no way to isolate the stressful environmental factors from the genetic background of each country. Scandinavians have both the genetic background of tall stature from their Viking ancestors and have a low corruption rate and high GDP.

Here, we focused on economic and social components of environmental stress, which are both a strengths and a limitation of the current study. We recognize the fact that environmental altitude and humidity may contribute to a child's growth and adult height. In Nepal and Peru, at the bottom of height spectrum, the influence of low oxygen pressure at high altitude has strong effect on growth along with the socio-economic environment (21). The statistical approach for analysis is to analyze all factors one by one. An additional limitation of this study is the fact, that we were limited by availability of the data on both growth and the environmental stress indicators; for only 71 countries we had both.

Child health and development are threatened by disasters, political violence, pandemics, and other adversities. Of these adversities, a stressful environment, as defined in this investigation as vulnerability to poverty, homicide, income inequality, corruption, unemployment, and pollution, threatens the health and well-being of many children. Here, we focused on child growth which culminates in the average final adult height at a national level, as a well-established indicator of stress (13, 15, 20, 22).

The results of our cluster analysis of the seven indices of environmental stress revealed that vulnerability is represented by (a) a suite of indices which we called QoL that comprises urban outdoor air pollution, life expectancy at birth, the CPI, and GDP per capita, and (b) a suite of indices which we called Social Factors which comprises homicide rate and economic inequality, as measured by the GINI index. We found that the most vulnerable nations are those of the developing world and those that have recently experienced conflict.

In this investigation, we used national averages in statural height, while recognizing this measure and its range are different in each country. Surprisingly, we found that between-countries variations are similar to those within countries. This comparison suggests that the range of 23–26 cm represents the saturation span for plasticity in height (2,9). We also found that a stressful environment exerts a greater effect on the height of males than that it does on females. Although women experience twice as many stressful events during their lives than men (23), we found that the impact of each and all of the stress indicators on male and female height is similar.

Here we show sexual dimorphism in growth vulnerability to stress even before reproduction. This finding is in line with evidence from humans and experimental animals: stress affects the behavioral, the endocrine, and the molecular responses of stress systems in the hypothalamus. Moreover, this effect presents itself in a clear sexual dimorphic way, with males being more vulnerable in their stress response (24).

The results of this study suggest a strong negative impact on growth of low GDP per capita, CPI, economic inequality, air pollution, and life expectancy, in that order. Milder negative effects on health and growth were found for air pollution, homicide rate, and unemployment. For understanding the mechanism of growth attenuation by stress, we define ‘homeostasis’ as the steady-state environment of the body that is threatened by stressors, and the “adaptive response” as those mechanisms which are activated to reestablish the steady-state (5). Up to a certain threshold of a stressor's strength and duration, the adaptive response can reestablish homeostasis without any cost to the individual. However, when a stressor cannot be entirely counteracted by the adaptive response and the homeostasis attained is suboptimal, it is associated with harm to the individual. Growth attenuation is a tradeoff adaptive response: a calorie used for growth cannot be used for combatting stress.

In this investigation, we have used national averages in height and environmental stress indicators while recognizing variations within countries. National averages comprise appropriate scales for information utilized by central governments for determining policies (25). Previous studies of national heights generally used indicators which were chosen subjectively by the authors and based on assumptions about the factors and processes leading to vulnerability, based on literature review, and intuitive understandings of human-environment interaction (26–29). The approach which we used in this study utilized indicators of vulnerability based on a conceptual framework in which risk is defined by established indicators for mortality outcomes (30).

Membership in the OECD reflects a minimum level of QoL. Since the QoL might have a different effect on child growth in developed and developing countries, we investigated the potential differential effect of QoL and social factors on child growth within member countries of the OECD. Although the effect of QoL on child growth was not statistically significant for member countries of the OECD, it is possible that this result is an artifact because of insufficient variation in QoL in developed countries. However, we found that the correlations with child growth were strongly significant when we measured more sensitive and more variable indicators of the social factor. Male and female heights are sensitive to the social factors of national economic inequality and homicide rate in all the countries and, specifically, in OECD countries.

In conclusion, we found that adult height, when used as a measure of a child's growth, is an indicator of a stressful environment in context with the genetic background and spatial factors. We also found that stressful QoL factors and the social factors exert a greater effect on the height of men more than that of women. Specifically, we found that the ranking of the indicators for a short stature are GDP per capita > the CPI > economic inequality > urban air pollution > life expectancy > homicide rate and unemployment.

## Data Availability Statement

Publicly available datasets were analyzed in this study. This data can be found here: http://data.worldbank.org/indicator/SP.DYN.LE0; http://data.worldbank.org/indicator/SL.UEM.TOTL.ZS; http://www.ncdrisc.org/d-height.html. The detailed information about data sources is provided in the Methods section.

## Author Contributions

AG: co-conceptualized and co-designed the study, reviewed, revised, and approved the manuscript. GM: co-conceptualized and co-designed the study, carried out the PCA statistical analysis, entered the results, reviewed, revised, and approved the study. ZH: conceptualized and designed the study, oversaw it's conduct and drafted the initial manuscript, reviewed, revised, and approved the study.

### Conflict of Interest

The authors declare that the research was conducted in the absence of any commercial or financial relationships that could be construed as a potential conflict of interest.
